# Gaze and movement adaptation in response to delayed robotic movement during turn-taking

**DOI:** 10.1038/s41598-025-17140-9

**Published:** 2025-09-30

**Authors:** Samantha Stedtler, Valentina Fantasia, Trond A. Tjøstheim, Ingar Brinck, Birger Johansson, Christian Balkenius

**Affiliations:** Department of Philosophy and Cognitive Science, Lund University, Lund, Sweden

**Keywords:** Human-Robot interaction, Delays, Gaze behavior, Robotic movements, Turn-taking, Neuroscience, Psychology

## Abstract

While delays in human-robot encounters can harm perceptions of competence, they can also enhance engagement and relatability, making timing a crucial factor in the design of robot behaviors for effective human-robot interaction. Previous research has primarily focused on how robots’ pauses or errors impact perceptions of competence, but it has not adequately addressed the complexity of timing in shaping social interactions. This study explored the effects of different delay conditions (no delay, short delay (4s), long delay (10s)) on human gaze behaviors and movements during a Tic-Tac-Toe game interactions with an Epi humanoid robot. We hypothesized that shorter delays would be interpreted as thinking pauses, increasing engagement, operationalized as directing gaze to the robot’s hand or face, while longer delays would lead to distractions, i.e. gazing away from the robot and perhaps searching for cues in other places. Gaze indicates attentional focus, a common proxy for engagement in HRI. The results, however, indicated that the no delay condition prompted the highest levels of participants gazing at the robot’s hand (’gaze to hand behavior’), with significantly more frequent gaze to hand behavior compared to both delay conditions. The duration of participants gazing at the robot’s face (’face gaze’) showed no significant differences, though trends suggested participants engaged more in face gaze in the no delay condition. Interestingly, during delay periods, participants spent significantly more time gazing at the robot’s face and hands, suggesting a monitoring response to unexpected pauses. Additionally, positive pre-interaction impressions of the robot were linked to higher gaze to hand behavior, while increased face gaze was associated with lower fluency ratings, indicating compensatory behavior during perceived interaction difficulties. These findings suggest that delays reduce overall engagement regardless of duration, while they might increase engagement temporally while the delays take place. The results also highlight the complexity of gaze behaviors in human-robot interaction and suggest that gaze patterns can indicate participants’ perceptions of the robot’s performance.

**Supplementary Information:**

The online version contains supplementary material available at 10.1038/s41598-025-17140-9.

## Introduction

A crucial aspect of a social robot is its capability to employ natural communication during user interactions^1^. The capacity to recognize and reference objects and people in the environment is frequently seen as a fundamental skill for robots to be able to engage in meaningful interactions. However, the role of time is often overlooked, even though it plays a significant role in making communication feel natural. This can be seen for instance in the binding role it takes to structure sequences and actions during an interaction and thus creating and altering meaning dynamically between co-participants^2,3^. Sociality involves anticipating others’ actions rather than merely reacting to them^4^. In this context, timing is critical for coordinating actions, engaging in social interactions, understanding intentions, and perceiving robots as collaborative partners^5–8^. From an interactional perspective, this temporality is observable in behaviors such as in the structure of reciprocal action sequences, or in how movements are seamlessly coordinated by co-participants^2^. Therefore, it seems like intentionality and sociality do not have to be represented internally but can be enacted in the way humans anticipate and respond to each other. Hence, actions are never ‘put on hold’ – their meaning simply changes with time passing between them. That is, refraining from acting is not perceived as neutral in ongoing interactions^9^. Given the inevitability of time lapses in Human-Robot interaction (HRI), understanding their influence on human behavior and perception is important. The same delay that might appear as an error in one situation can feel appropriate in another, and the interpretation of an action can shift based on the timing of its execution. This ambiguity raises the question of how we should account for the intricate relationship between timing and social interaction when designing robot behaviors. One way to investigate this is by measuring the perceived social nature of the robot and the interaction itself, during and after delays.

## Related work

A delay can be interpreted as either a technical error (i.e., a divergence from expected behavior^10^) or as an intentional act of a social agent. The ability of a robot to convey understandable and predictable intentions is essential for effective interaction^11,12^. For instance, Pelikan and Hofstetter^9^ found that explaining delays within the immediate context makes them accountable and easier for human partners to comprehend.

The perception of a robot’s errors is closely linked to its perceived social agency and human-likeness. For instance, collaborative performance has been shown to decrease after robot errors, yet participants’ positive emotions toward the robot seem to grow^13^. The latter might suggest that errors make the robot seem more human and relatable. Conversely, another study found that a robot was perceived as less human-like after making errors^14^. This discrepancy indicates that more research regarding the context and impact of erroneous behavior in HRI has to be conducted to fully understand their effects. The effect of both errors as well as recovery strategies have been shown to vary between human versus robot employees when it comes to trust and forgiveness^15^. Thus, whether a robot is more human- or robot-like should matter for error-tolerance and negative effects of the errors too. Mirnig et al.^16^ argue that when a robot behaves unexpectedly, people are more likely to perceive it as a social actor. Perceived intentionality is an essential aspect of sociality, since humans often apply the intentional state to interpret others’ actions, viewing them as the result of mental processes^4^. Whether this intentional stance – or design stance as more commonly adapted towards technology – is taken towards robots, depends amongst other things on the types of errors (human-like vs. machine-like) that are committed. Previous studies have distinguished between technical errors and social norm violations^10,17^.

The timing and nature of delays can also convey specific information. Varying a robot’s action speed can express attributes like confidence, while pauses can negatively affect perceptions of competence and positively influence perceptions of an object’s weight^18^. Delays can also have benefits, such as fostering user affinity or providing necessary time for comprehension^8,12,19^. For example, participants interacting with a route-directing robot favored a pattern with pauses, even if they were unusually long, because it provided sufficient time to process the robot’s responses^19^. Therefore, avoiding immediate responses can help align with a natural conversational rhythm and give the impression that the robot is processing information^18^.

When talking about robot failures, the concepts of trust and fluency are central as well. Trust in a robot can be viewed as the user’s explanation for a reliable sensory exchange^20^. The timing of failures can dynamically affect this trust; unreliability early in an interaction has a more lasting negative impact than later failures^21^.Hoffman and Breazeal^12^ suggest that interactional fluency, closely related to user comfort, should be emphasized along with task efficiency in human-robot interaction (HRI) studies. Fluency is typically perceived unconsciously through the alignment of expected and actual outcomes, meaning that unexpected pauses can disrupt this fluency^22^. These findings suggest a hypothesis: small delays may be perceived as human-like and enhance a robot’s sociability, whereas long delays are more likely to be seen as technical breakdowns. Consequently, a robot that is always perfectly punctual might be perceived as too predictable and less socially engaging. Another aspect of being with a teammate is the readiness to adjust one’s own actions to theirs^23^. The Temporal Behavior Matching Hypothesis posits that humans actively synchronize with the dynamics of robots during HRI^24^. This was supported by Kose-Bagci et al.^25^, who found that participants adapted their action timing to match a robot’s rhythm in a drumming task. Gaze is another critical nonverbal behavior for coordinating social interaction and signaling intent^26,27^. In conversation, gaze helps manage turn-taking and convey attentiveness, making it a potential behavioral indicator of fluency^28^. During object manipulation, eye gaze typically precedes an action, thereby signaling goals and improving the predictability of an agent’s intentions^29,30^. In HRI, eye gaze is extensively studied for its role in communication, engagement, and error detection^26^. Directed gaze and mutual facial gaze are indicators of engagement^31^. For example, social movements performed by a robot were shown to increase participants’ gaze toward its hands and face^32^. Specific gaze patterns can also indicate unexpected behavior or pauses, such as looking back at the robot’s face when a response is anticipated but not received during a turn-taking interaction^33^.

### Current research

This study examined the entanglement of timing of movement with sociality by analyzing the effect of delays (10 seconds and 4 seconds) on participant behavior and correlations of behavior and self-reported perception of the interaction. The specific robot used, participant task setup, and study hypotheses are detailed in the Methods section (see Fig. 4, 5 and 6). While we acknowledge that a 10-second delay is quite prolonged, our aim was to observe reactions to pauses that exceed what is typically considered natural. The 4-second delay, on the other hand, may still be noticeable but is closer to the rhythm of natural human-human interactions. Delays in both human-human and human-robot interactions seem beneficial when they align with expectations or fit into a social script, but negative effects if they are unexpected or excessively long. We anticipate negative responses in terms of decreased engagement (indicated by gaze) and ratings for the long delay condition. For the short delay condition we hypothesize that participants interpret delays as thinking pauses where the robot considers its next move, resulting in more engagement and higher perceived sociality. In addition, previous research has shown that people’s initial attitudes toward robots can affect their perception of robotic behavior, suggesting that individuals with more favorable attitudes might be less likely to lose trust or perceive a decline in fluency during a delay^22^.

In a previous study, we analyzed self-report questionnaires in terms of fluency, as well as key social attributes such as anthropomorphism, animacy, and likability^34^. We did not find any significant differences between different lengths of delays, concluding that there might be less conscious ways in which this kind of behavior manifests.

The aim of the current study was to investigate the influence of time delays in robotic movements. In many HRI studies, it is common to use self-report measures and base recommendations for future robotic features on the reported results. However, it is possible that there are subtle changes which could not be captured by questionnaires. Therefore, we used behavioral features to see whether they indicate different reactions to delays and whether these features are connected to different subjective ratings. We used measures of gaze behavior in terms of gaze to hand behavior (participant gazing at the robot’s hand), face gaze (participant gazing at the robot’s face), and gaze away as indexes of coordinating and negotiating socially relevant processes during turn-taking between participants and the robot (drawing inspiration from Bakeman and Quera’s work on observational methods for studying social interactions^35^). We analyzed these behaviors both in terms of general duration and frequency of occurrence, but also in terms of patterns, and whether these patterns change as a result of a delay. We aimed to answer the following research questions:

- *Q1* Do delays in the robot’s playing actions influence the human participants’ behavior, as indexed by a) changes in the overall gaze behavior? b) occurrence and changes of gaze patterns during the delay?
- *Q2* Do participants adjust the timing of their playing actions to the robot’s timing over time, and is this more likely for certain conditions?
- *Q3* Is there a correlation between gaze behavior and perception of the robot’s social attributes (e.g. fluency, anthropomorphism, likability, intelligence etc.)?

Regarding *Q1a)*, we hypothesised that the long delay condition would lead to negative responses, reflected in decreased engagement (as indicated by gaze behavior). Specifically, we expected increased monitoring of the robot (i.e., gazing at it) during delay periods and more gaze away behavior, indicating the robot is perceived less as a social partner. In contrast, in the short delay condition, we expected delays to be interpreted as natural thinking pauses, leading to higher engagement and perceived sociality, reflected in more sustained gaze at the robot and less gaze away behavior. As humans tend to show specific gaze behaviors when detecting problems or unexpected events, we expected changes in participant gaze during delay periods *(Q1b))*. Specifically, we expected more looking at Epi’s face and actions during delays to monitor the situation and understand what was happening. Concerning **Q2**, we hypothesized that participants would gradually adjust their timing to align with Epi’s, especially in the no delay and short delay conditions, which better resemble natural human interaction. This expectation is based on research showing that people synchronize their movements with agents they see as competent social partners^23^. For **Q3**, we hypothesized that participants who perceived the interaction as more fluent would look less frequently at the robot, needing less monitoring. At the same time, participants rating the robot high in intelligence or anthropomorphism might look more at its hands, treating it as an intentional agent; those with lower ratings might gaze away more, seeking alternative explanations (e.g., from the researcher).

## Methods and materials

### Participants

In this study, seventeen participants (11 female, 6 male), aged between 20 and 34, were recruited from various academic disciplines among the students and staff at (anonymised for blind review) University. All participants provided informed consent before the experiment and were compensated for their involvement.

**Fig. 1:**
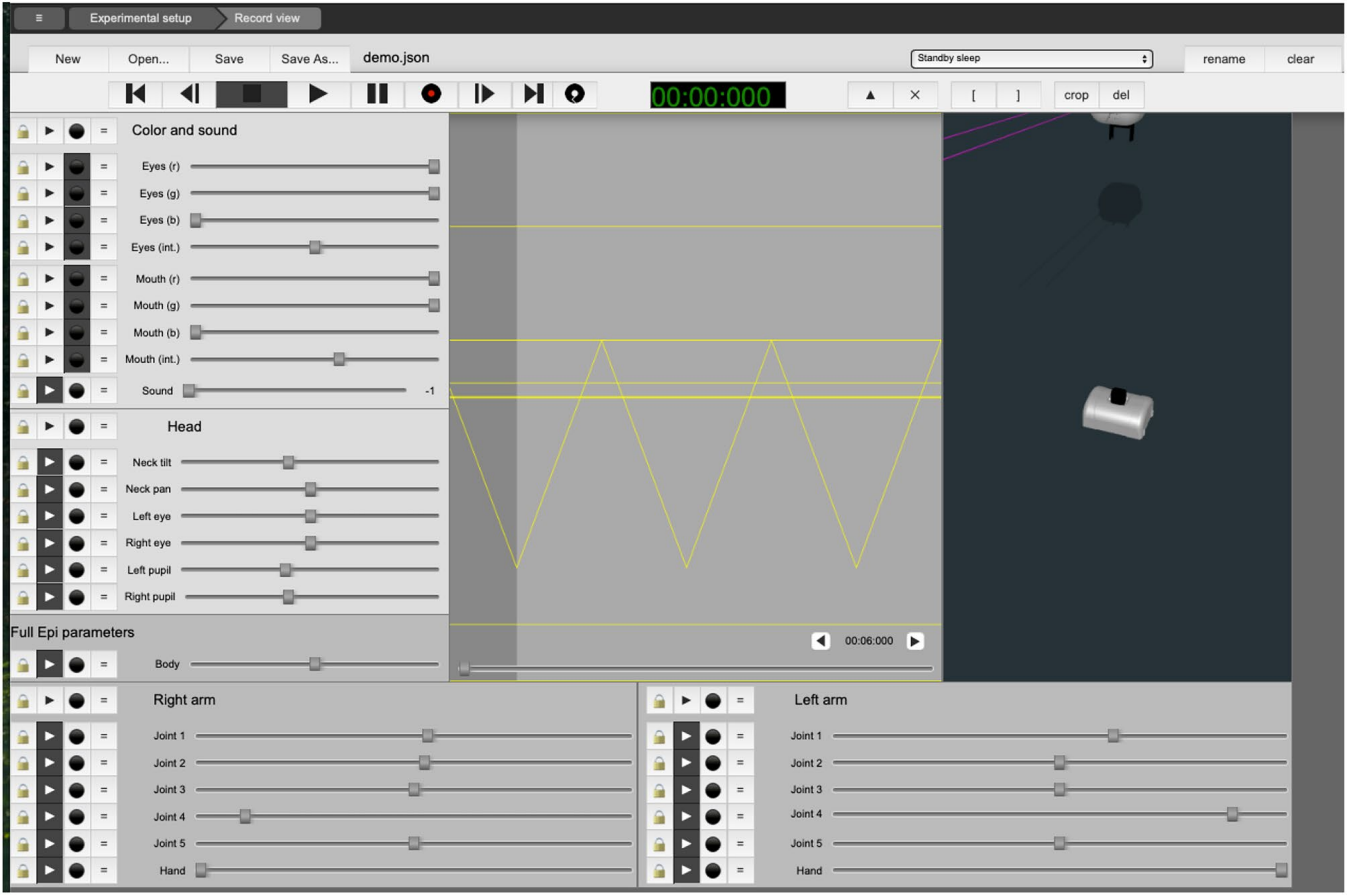
The Ikaros WebUI running in a web browser, showing the recording view with system controls to execute movement.

**Fig. 2:**
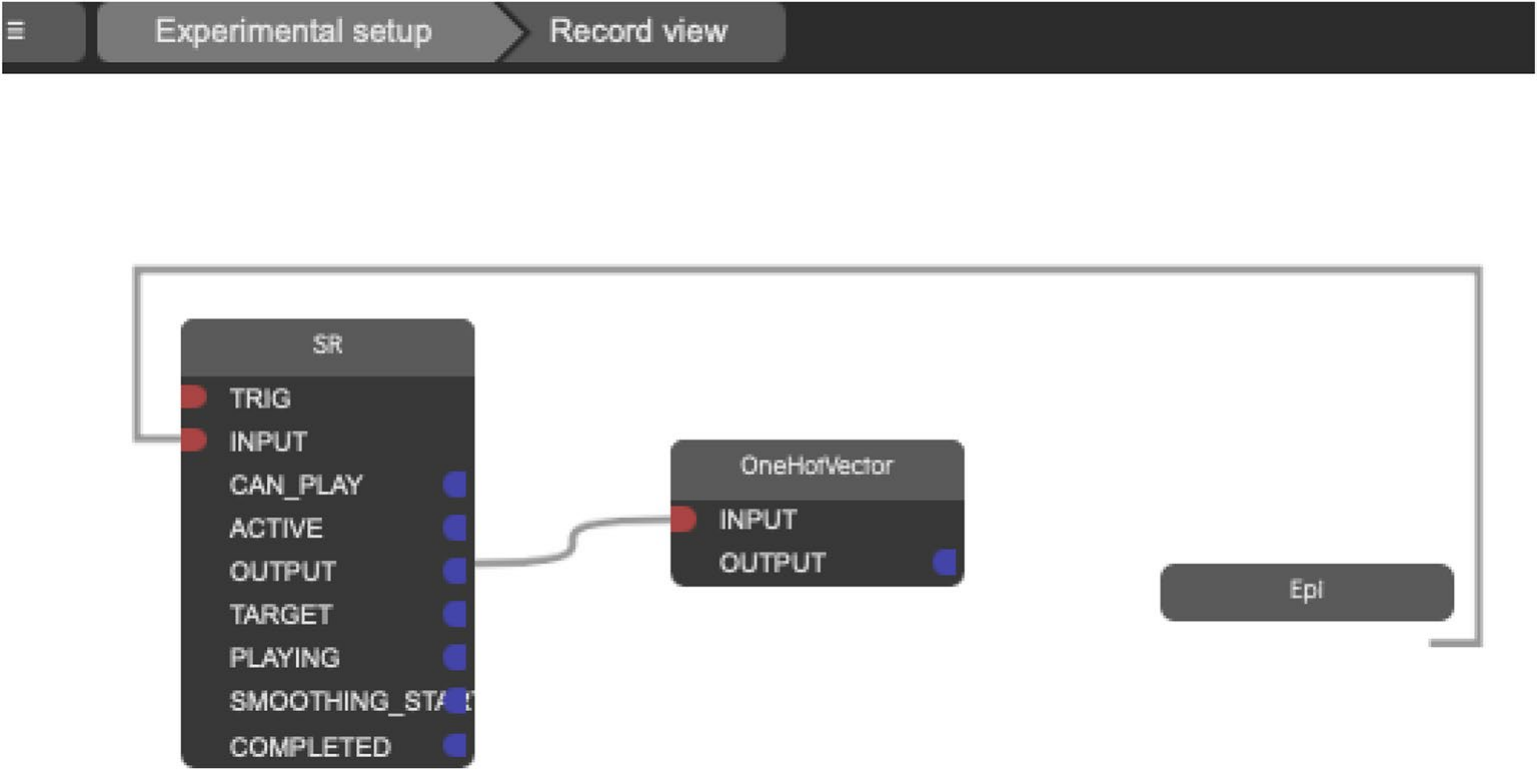
The Ikaros WebUI, showing the module structure and connections between the modules.

### Design and procedure

**Fig. 3:**
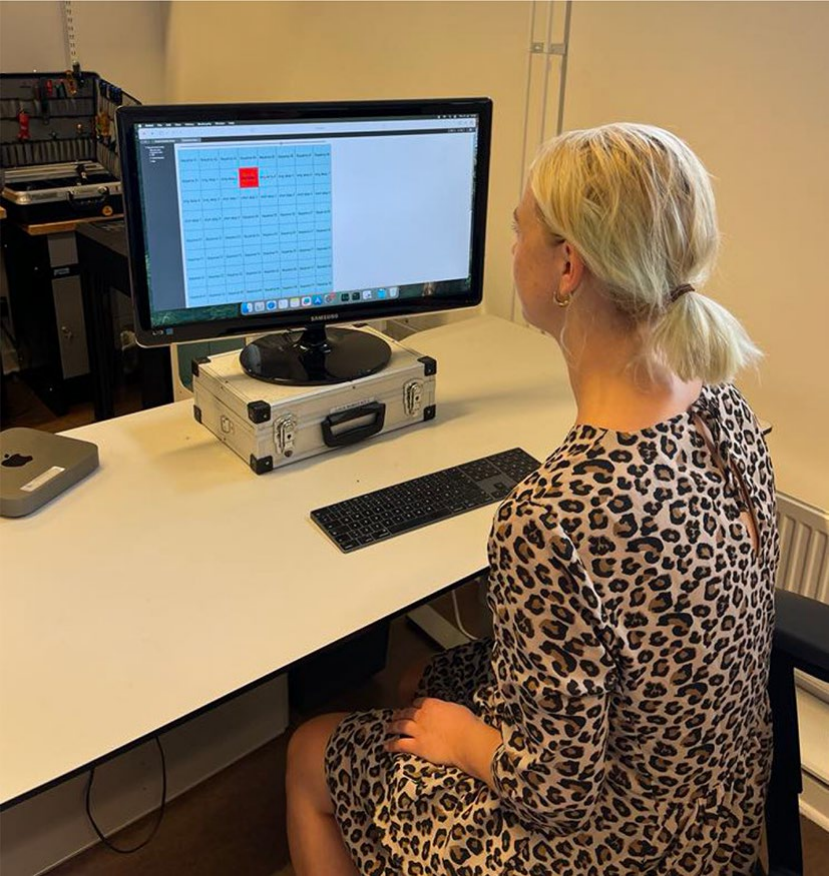
Researcher/ human operator choosing the movement sequences on the playback menu in Ikaros.

We utilized the humanoid robot Epi, developed by the (anonymised for blind review) Cognitive Robotics team, and employed remote control (also called Wizard of Oz methodology, WoZ) using Ikaros software^36^ to operate the robot^37^. In the WoZ paradigm, a human remotely operates the robot using buttons or joysticks. In our case, we used the Ikaros software to record movements for picking up and placing markers on the game board (Figs.1 and 2). Recording was done by manually guiding the robot’s arm through the necessary movements. Each movement was then saved to a specific GUI button, and were then triggered by the human operator as appropriate during game play (Figs. 3 and 5). The system latency between the operator triggering a movement and the robot’s action execution was measured to be less than a second, thus estimated to have a negligible effect on the interaction process.

**Fig. 4:**
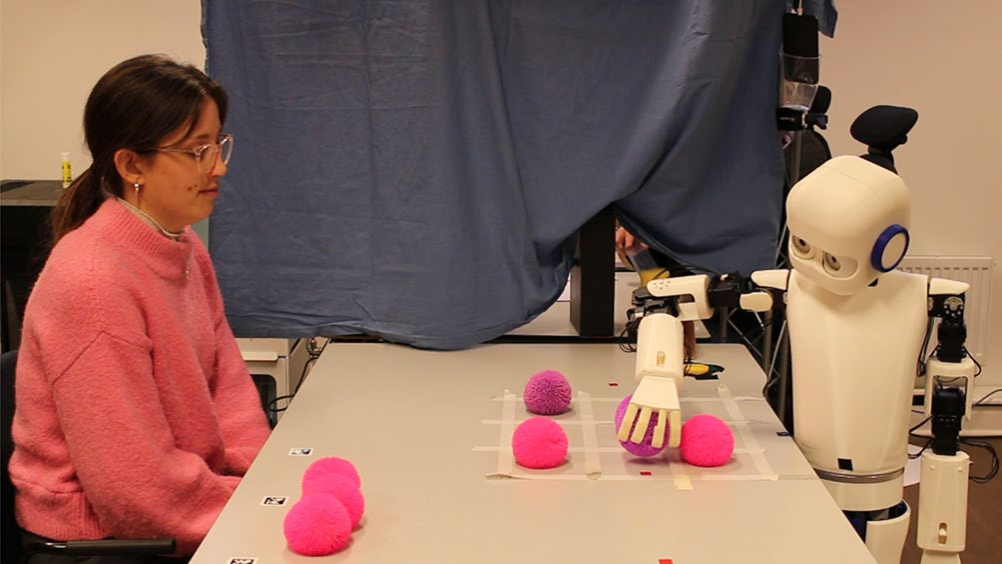
Task setting, showing participant (P) playing Tic-Tac-Toe against Epi (E). Written consent to publish the picture was obtained from the participant.

**Fig. 5:**
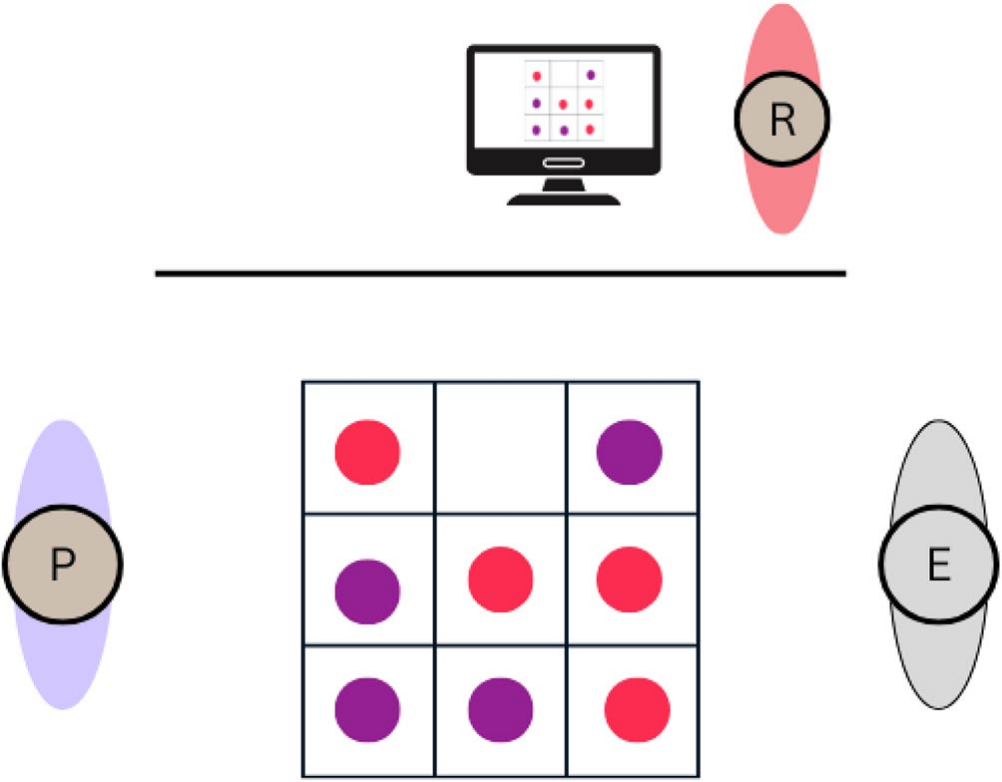
Task setting from above. Researcher (R) is sitting behind a curtain and controlling which movement sequence Epi will use next.

Participants were welcomed by the experiment leader and provided their demographic details along with their initial attitudes towards the robot using the NARS questionnaire^38^. They then completed the Godspeed Questionnaire, which assessed their perceptions of the robot’s intelligence, animacy, anthropomorphism, likability, and safety before engaging in the interaction^39^. Participants engaged in eight rounds of Tic-Tac-Toe against Epi while being recorded on video. The movements displayed by Epi were pre-recorded and individually chosen by the experiment leaders during the study. Throughout the interaction, the robot did not display any gaze behavior, which prevented it from using gaze cues to compensate for the long delays or to signal where it intended to place the ball. This was an intentional design choice to reduce complexity and to ensure that any measured effects are due to the delay conditions and not due to other factors. Following this interaction, the participants were asked to complete the Godspeed Questionnaire once more, in addition to Hoffman’s Fluency Scale and the Trust Perception Scale^40,41^. Tic-Tac-Toe was chosen as a task because it is simple enough to allow focusing on movement effects rather than complex game strategies. The game is very easy and is often used in social contexts, which is aligned with our aim to investigate the influence and possibilities of Epi as a social agent^42^. The setup involved Epi being positioned on one side of a table with the Tic-Tac-Toe grid, composed of two horizontal and two vertical lines, clearly marked on the table surface. Participants were seated opposite Epi. The game was played by placing colored balls on the grid, with the objective of forming a row of three balls vertically, horizontally, or diagonally to win each round (Figs. 4 and 5).

### Experimental conditions

**Fig. 6:**
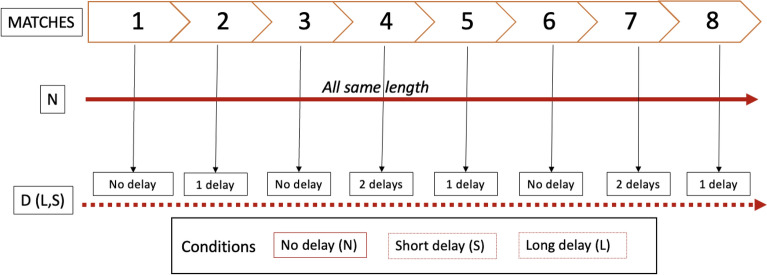
Visualization of the temporal sequence of matches across experimental conditions. Each colored block represents one match. The figure illustrates the order and amount of delays occurring in Epi’s movements over the course of the interaction.

This study utilized a between-group design, with participants randomly assigned to one of three conditions: no time delay (N, *n*=6), short 4-second delay (S, *n*=6), or long 10-second delay (L, *n*=5). These time windows were selected based on previous exploratory findings suggesting that participants were relatively tolerant of even longer delays and barely noticed short ones. This indicates that the commonly cited “tolerable” window of 2 seconds may, in fact, be too short (in HCI research, the so-called “two-second rule” has served as a general guideline for acceptable system response times)^43,44^. We chose 4 seconds as it lies near the midpoint between 0 and 10 seconds, while still being close to what might be considered normal during an interaction due to the two-second rule, thus making it a compromise between these two considerations. Initially, all participants completed one match without any time delay. In subsequent matches, Epi’s behavior varied according to the assigned condition, with the robot either continuing without delays (no delay condition= N), or introducing short delays (S), or incorporating longer delays (L) (Fig. 6). During a delay, Epi would halt its movement just before placing the ball on the grid, momentarily hovering its hand above the table. The first match was left without any delays to allow participants to become familiar with the flow of the game. Delays were introduced during the second, fourth, fifth, seventh, and eighth matches to reduce predictability and prevent participants from anticipating when a delay would occur. In three of the delayed matches, only one delay occurred: at the beginning of the second match, and during the middle of the fifth and eighth matches. In the remaining two matches (the fourth and seventh), there were two delays, one at the beginning and one at the end of each match. While the sequence of delayed matches was fixed across participants, the exact timing of each delay within a match varied. The duration of each delay depended on the participant’s assigned condition group (none, 4, or 10 seconds). We did not counterbalance the delay order across participants in order to maintain consistency in task progression and to ensure that any observed effects could be attributed to the delay duration rather than differences in sequence complexity. As with all movement sequences in the experiment, those containing delays were pre-recorded using the movement module of the Ikaros platform and manually triggered by the researcher during the game. Because the sequences were pre-recorded, movements were identical across participants for each sequence with the same characteristics (i.e., delay length and ball end-location).

### Measures

#### Behavioral measures and coding

To address our research questions, we identified and measured the following participant behaviors: gaze to hand behavior, face gaze, gaze away as well as the length of turns. The delays in Epi’s movements were also coded in order to temporally and sequentially relate their occurrence to the occurrence of participant behavior. The duration and relative frequency of each behavior were coded on a second-by-second basis, with each second coded only once by an observer who was blind to the experimental hypotheses and conditions. For this coding process, we utilized Datavyu (datavyu.org), a video analysis software that facilitates the creation of detailed annotations on video and audio resources. To ensure reliability, a second blind observer independently coded 25 % of the participants’ data. Inter-observer agreement was assessed using the “agree” function in the irr package in R, as well as Cohen’s Kappa coefficient. The reliability of the coding was high across all behaviors, with a Cohen’s Kappa coefficient of 0.93 for all gaze behaviors. Specific agreement rates were 86.73% for gaze to hand behavior, 93.93% for face gaze, and 79.18% for gaze away, all with p < 0.001. In the following, we will describe how the categories of gaze behaviors of interest were defined in the coding scheme.

- *Category 1: Gaze to hand behavior* Gazing towards Epi’s hand/ arms (eyes pointing towards Epi’s hand/arm, head might be slightly tilted down, following the movement of Epi’s hand/arm with the eyes or head posture). Onset with the first frame when eyes are pointed towards Epi’s hand and offset with the first frame when eyes are no longer pointed towards Epi’s hand.
- *Category 2: Face gaze* Gazing towards Epi’s eye or face. Onset with the first frame when eyes are directed towards the other player’s face and offset with the first frame when eyes are no longer directed towards the other player’s face.
- *Category 3: Gaze away* Gazing towards a distant, undefined point in the room. Onset with the first frame when gaze shifts from face/hands towards a distant, undefined point in the room and offset with the first frame when participant directs their gaze again at Epi’s hand, Epi’s face or the playing grid.

Based on the gaze behaviors we coded according to the definitions provided above, we calculated two measures: gaze duration and relative frequency of each gaze type. Gaze duration was measured by summing the duration (in milliseconds) of each instance of a given gaze behavior, from its onset to its offset, within each match. Relative frequency was calculated by dividing the number of occurrences of each gaze behavior by the total number of gaze behaviors recorded during the same match. These metrics were chosen because they capture both the temporal and frequency-based aspects of gaze behavior, ensuring robustness of our chosen gaze-measures.

#### Self-report questionnaires

*Pre-Interaction Questionnaires*: Before the interaction, participants completed a series of questionnaires to capture their subjective experiences. They began by providing demographic information and then completed the Negative Attitudes Towards Robots Scale (NARS). The Godspeed Questionnaire, using a 5-point Likert scale, was used to assess participants’ perceptions of anthropomorphism, animacy, intelligence, likability, and safety. *Post-Interaction Questionnaires*: Following the interaction, participants were asked to complete the Godspeed Questionnaire again. The Hoffman Fluency Scale (7-point Likert) was used to evaluate interaction fluency, while the Trust Perception Scale measured levels of trust. As a manipulation check, participants were also asked if they believed Epi made any errors during the interaction.

### Ethical considerations

All methods in this study were conducted in accordance with relevant guidelines and regulations, including the Declaration of Helsinki, the Swedish Act Concerning the Ethical Review of Research Involving Humans, and the EU General Data Protection Regulation (GDPR). Participants provided written informed consent before taking part in the study. All experimental protocols were reviewed and approved by the Swedish Ethical Review Authority (Etikprövningsmyndigheten). Specified written consent to include an image (Fig. 4) that could potentially lead to the identification of a study participant has been obtained from the subject. The consent explicitly permits the publication of the image in a scientific publication (print or online).

## Results

### Influence of delays on gaze in human-robot interaction

#### Q1a): Is there an effect of the robot’s delay on the human participant, as indexed by changes in the overall gaze behavior?

Descriptive results of gaze duration are summarized in Table 1. Overall, gaze to hand behavior was highest in the N condition (Mean=40.6%, SD=13.1%) and lowest in the S condition (Mean=28.8%, SD=14.3%). Face gaze was highest in the L condition (Mean=9.82%, SD=8.38%) and lowest in the S condition (Mean=7.16%, SD=7.36%). Gaze away was highest in the L condition (Mean=0.86%, SD=1.6%) and lowest in the S condition (Mean=0.26%, SD=0.56%). Since the distribution of data violated the assumption of normality, we performed a log transformation with a subsequent two-way ANOVA (with Condition and Match number being independent variables) in R. The results showed no effect of match number (i.e. changes over time) on duration of any of the gaze behaviors (see Table 2; F-value(7, 118)= 1.36, *p*=0.23). There was a significant effect of condition on gaze to hand behavior duration (F-value(2, 118)= 9.28, *p*<0.001, partial $\eta ^2$= 0.14). For gaze frequency, there was a significant effect of condition on both gaze to hand behavior (F-value(2,126)= 7.47, *p*<0.001, partial $\eta ^2$= 0.12) and gaze away behavior (F-value(2,126)= 3.28, *p*<0.05, partial $\eta ^2$= 0.05; see Table 3).

**Table 1:** Means and standard deviations for Gaze Durations in Percentage (Gaze to hand behavior, Face Gaze, and Gaze Away) by Condition.

Variable	No delay	Short delay (4s)	Long delay (10s)
Gaze to hand behavior	40.6 (13.1)	28.8 (14.3)	31.1 (10.1)
Face gaze	8.76 (7.63)	7.16 (7.36)	9.82 (8.38)
Gaze away	0.66 (1.31)	0.26 (0.56)	0.86 (1.60)

**Table 2:** Two-way ANOVA results for log-transformed *durations* across gaze types.

Gaze type	Effect	Df	F value	p-value	Partial $\eta ^2$
Gaze to hand behavior	Match number	7	1.360	0.23	–
	Condition	2	9.275	<0.001 ***	0.136
	Residuals	118	–	–	–
Face gaze behavior	Match number	7	0.964	0.46	–
	Condition	2	2.635	0.08	0.041
	Residuals	122	–	–	–
Gaze away	Match number	7	1.227	0.31	–
	Condition	2	2.930	0.06	0.113
	Residuals	46	–	–	–

Significance codes: *** $$p < 0.001$$, ** $$p < 0.01$$, * $$p < 0.05$$, . $$p < 0.1$$

**Table 3:** Two-way ANOVA results for log-transformed *frequency* across gaze types.

Gaze type	Effect	Df	F value	p-value	Partial $\eta ^2$
Gaze to hand behavior	Match number	7	0.870	0.53	–
	Condition	2	7.465	<0.001***	0.106
	Residuals	126	–	–	–
Face gaze behavior	Match number	7	0.271	0.96	–
	Condition	2	1.365	0.26	0.021
	Residuals	126	–	–	–
Gaze away	Match number	7	1.938	0.07	–
	Condition	2	3.283	0.04 *	0.050
	Residuals	126	–	–	–

Significance codes: *** $$p < 0.001$$, ** $$p < 0.01$$, * $$p < 0.05$$, . $$p < 0.1$$

**Fig. 7:**
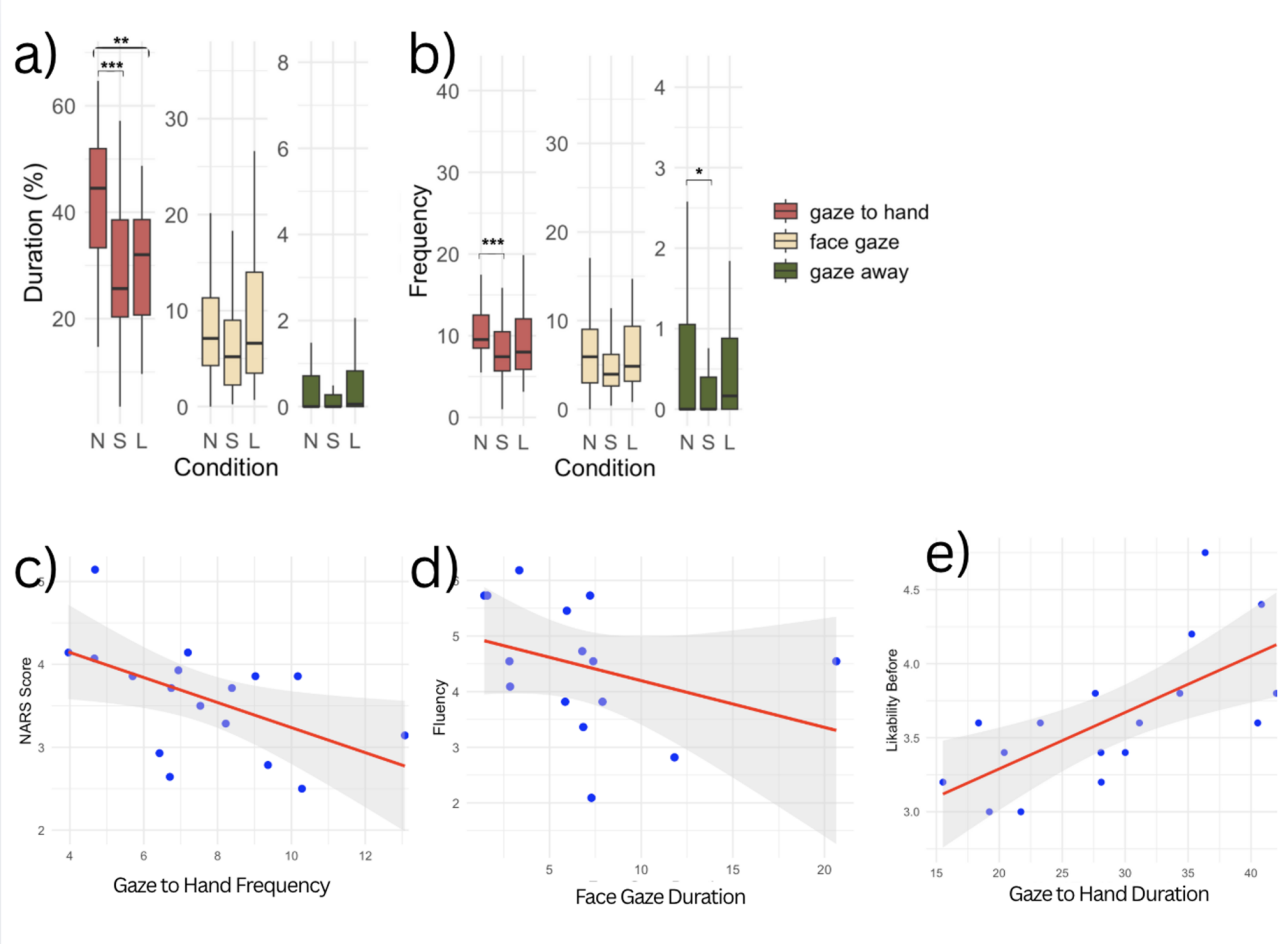
**a** Boxplot comparing Gaze duration in percentage across conditions (N=no delay, S= short delay, L= long delay). * *p*<0.05, ** *p*<0.01, *** *p*<0.001. **b** Boxplot comparing Gaze frequency (mean) across conditions (N=no delay, S= short delay, L= long delay). **c** Spearman’s rank Correlation between Gaze to hand behavior Frequency and NARS **d** between Face Gaze Duration (%) and Fluency e) between Participant Gaze to hand behavior Duration (%) and Likability.

We performed a post hoc pairwise-comparisons between the means of groups using the Tukey Honest Significant Differences (HSD) test in R (see Appendix, Table 1). We found a significant difference between conditions N and L (N > L, p<0.05). We also found a significant difference between conditions S and N (S < N, p<0.001, see Fig. 7a). There was no significant difference between conditions S and L (p=0.21). Thus, it seems like there was a higher duration of gaze to hand behavior present during the no delay condition than in either of the delay conditions.

The HSD test for relative frequency showed that the no delay condition exhibited significantly more gaze to hand behavior than the short delay condition (Fig. 7b), however there was no significant effect between no delay and long delay (p=0.13, see Appendix, Table 2).

For face gaze duration, the effects of condition were not significant, the same was the case for relative frequency (see Appendix, Table 2). For gaze away duration, we found no significant effects for condition (0.04, p = 0.78), however there was a significant effect of match number, indicating a slight increase in gaze away behavior over time (0.12, p<0.001). The post hoc test showed a close-to significant difference between the short delay and long delay condition (p=0.08), with gaze away duration being almost significantly lower for the short delay condition. In terms of relative frequency, there was a significant effect of condition (p<0.05), with the post hoc showing a significant difference between S and N (S < N, p<0.05) and with a trend towards significance between S and L (S < L, p=0.07). Thus, gaze away is lower in the short delay condition than in both the no delay and the long delay condition.

For completeness, we conducted both a post hoc power analysis and a complementary Bayesian multilevel regression analysis (see appendix). Differences in mean frequency between conditions varied in magnitude, with observed effect sizes ranging from small ($|d| \approx 0.07$) to large ($|d| \approx 1.30$). Nevertheless, the wide 95% confidence intervals associated with these effect sizes (e.g., for Gaze to Hand: N vs. S, 95% CI = [-0.12, 2.71]) highlight the considerable uncertainty surrounding the true population effect sizes –a direct consequence of the limited sample size. Post hoc power analysis (PPA) for observed effects demonstrated low power across all comparisons (ranging from 0.05 to 0.53), indicating that the study was indeed somewhat underpowered to reliably detect effects of the observed magnitudes (noting that PPA has its own limitations; see, e.g.,^45^). We applied Bayesian multilevel regression to assess differences in gaze duration inside versus outside delay periods. Models were fit using a Student’s-t distribution to mitigate the influence of outliers. For gaze to hand and gaze away, simpler models without participant-level random effects performed best. For gaze to face, a model including participant random effects provided the best fit (see Appendix, Table 3). The Bayesian analysis showed negative difference scores with P(Diff < 0) $\ge$ 0.97 for the face gaze category and no differences for the gaze away category. The gaze to hand behavior measure showed a more dynamic pattern with matches 2, 7, and 8 having P(Diff < 0) $\ge$ 0.9 while matches 4 and 5 had P(Diff < 0) of 0.91 and 0.55 respectively (see Appendix, Table 4).

**Table 4:** Effect sizes and post hoc power for pairwise comparisons of mean frequency.

Frequency set	Comparison	Effect size (*d*)	CI (lower)	CI (upper)	Post Hoc Power ($\alpha =0.05$)
Gaze to hand	N vs S	1.30	− 0.12	2.71	0.53
Gaze to hand	N vs L	0.49	− 0.90	1.88	0.12
Gaze to hand	S vs L	− 0.42	− 1.81	0.96	0.10
Gaze to face	N vs S	0.49	− 0.82	1.79	0.12
Face gaze	N vs L	0.17	− 1.20	1.55	0.06
Face gaze	S vs L	− 0.34	− 1.72	1.04	0.08
Gaze away	N vs S	0.69	− 0.63	2.02	0.19
Gaze away	N vs L	− 0.07	− 1.44	1.30	0.05
Gaze away	S vs L	− 0.85	− 2.28	0.58	0.26

#### Q1b): Is there an effect of the robot’s delay on the human participant, as indexed by occurrence and changes of gaze patterns during and after the delay?

We applied the Friedman test to compare the durations of each gaze behavior inside the delay periods and outside the delay periods. Comparisons were not calculated for the first, third, and sixth match, since these matches did not contain any delays. No significant differences were found for this measure (Friedman $\chi ^2$ = 4.24, df = 4, p = 0.3745), indicating that there was no consistent global effect of the delay on gaze behavior across all matches. To further explore possible localized effects, we applied the Wilcoxon signed-rank test to compare the durations of each gaze behavior inside and outside the delay periods for the individual matches (see Table 5). We found significant differences in gaze to hand (overall $p < 0.01$) and face gaze behavior (overall $p < 0.01$), with both gaze behaviors being more present in participants’ behavior during delays. Regarding gaze to hand behavior, the strongest differences were present in the second ($p < 0.01$) and the seventh ($p < 0.05$) match. Concerning face gaze, the difference was strongly significant in all matches. Gaze away was not significantly different between the periods inside and outside of delays. These results suggest that while there is no overall effect of delay periods on gaze behavior across all matches, there are match-specific differences indicating that delays may affect gaze behavior at a more local level. This trend was also confirmed using a complementary Bayesian analysis (see Appendix, Fig. 1 and Table 4). The posterior summaries indicated robust effects of delay for gaze to face (e.g., Match 2: M = –19.93, 95% CrI [–33.55, –6.06], P(Diff < 0) = 1.00) and gaze to hand (e.g., Match 2: M = –24.50, 95% CrI [–40.21, –8.58], P(Diff < 0) = 1.00), consistent with the Wilcoxon results. For gaze away, no credible difference between delay and non-delay periods was observed. These findings strengthen the evidence for gaze behavior changes during robot delays and account for match-specific and participant-level variability (see Appendix, Tables 3, 4 and 5 and Fig. 1).

To check for sequential patterns, we calculated the probability for each gaze behavior to occur as the first behavior during the delay, and to occur as the second gaze behavior (see Table 6). We did not include gaze away behavior in this analysis since there were too few instances during the delays. The probability to occur as the first gaze behavior was almost the same for gaze to hand behavior and face gaze, as was the probability to occur as the second behavior. We also calculated the average transition time between the different gaze behaviors. The fastest transition happened after face gaze to gaze to hand behavior while the longest time to transition happened from gaze to hand behavior to face gaze. The differences in transition time were however not significant $\chi ^2 = 1.52$ (df = 3, p = 0.68).

**Table 5:** Comparison of gaze behavior between periods outside the delays and inside the delays across matches. reporting mean (Duration in %), standard deviation and V-value and p-value of Wilcoxon signed-rank test. * *p*<0.05, ** *p*<0.01, *** *p*<0.001.

	Gaze to hand behavior		Face gaze		Gaze away	
	In	Out	In	Out	In	Out
Second Match	47.61 (20.25)	22.37 (8.39)	23.07 (19.13)	3.45 (2.57)	0 (0)	0.12 (0.23)
	V = 3, p-value < 0.005**		V = 4, p-value < 0.007**		V = 6, p-value = 0.18	
Fourth Match	27.86 (20.63)	17.78 (10.75)	29.93 (25.78)	4.55 (3.52)	1.41 (2.66)	0.28 (0.36)
	V = 16, p-value = 0.14		V = 3, p-value < 0.005**		V = 6, p-value = 0.4	
Fifth Match	27.58 (25.19)	22.37 (14.36)	19.30 (19.64)	3.51 (4.08)	0.44 (1.39)	0.24 (0.44)
	V = 30, p-value = 0.85		V = 5, p-value = 0.02*		V = 6, p-value = 0.86	
Seventh Match	34.96 (16.75)	18.48 (10.55)	28.59 (23.02)	2.42 (3.81)	3.32 (6.15)	0.49 (0.86)
	V = 10, p-value = 0.04*		V = 1, p-value < 0.002**		V = 9, p-value = 0.23	
Eighth Match	38.71 (30.54)	21.63 (14.58)	26.89 (20.80)	6.21 (5.09)	0.68 (2.26)	0.34 (0.46)
	V = 16, p-value = 0.15		V = 3, p-value < 0.005**		V = 10, p-value = 0.59

**Table 6:** Table showing probabilities and transition times with standard deviations for gaze transitions.

Gaze transition (first-second)	Probability (%)	Gaze transition time (ms)
Hand-hand	15.2 (11.3)	840 (992)
Hand-face	30.3 (10.3)	1737 (3645)
Face-hand	31.8 (10.2)	688 (1194)
Face-face	13.6 (11.4)	913 (813)

### Movement adaptation in turn-taking

#### Q2: Do participants adjust the timing of their actions to the robot’s timing over time, and is this more likely for certain conditions?

First, we checked whether there was an overall correlation between the course of matches and difference in turn-length. Mean turn lengths in the different conditions were 4591 ms (SD = 1505 ms) for the no delay condition, 6051 ms (SD = 2235 ms) for the short delay condition and 5291 ms (SD = 2197 ms) in the long delay condition. Thus, variability between subjects was quite high. We used the Spearman’s correlation test since the data was not normally distributed and was not eligible for log transformation^46^. To see whether participants adapt to Epi’s timing with their playing actions, we calculated the difference in average turn-length for each participant during each match. For difference in turn-length, the overall correlation with match-progression was $\rho = 0.24$, and for the different conditions $\rho = 0.13$ (N), $\rho = 0.33$ (S) and $\rho = 0.38$ (L). This suggests that there is a slight tendency for difference in turn-length to increase with match numbers, but the relationship is weak. It also seems like correlation was weakest for the no delay condition and strongest for the long delay condition, meaning that the difference might have changed most over time in the latter.

While we did not find strong correlations, the correlations seemed to be influenced by the condition. Therefore, we conducted a Kruskal-Wallis rank sum test on turn length to determine whether there is a difference between conditions. We found a result of $\chi ^2 = 13.98$ (df = 2, p<0.001). We used the Dunn’s procedure as a post hoc test and found significant differences between condition N and S (z = -3.72, p < 0.001). We found no significant overall-difference between conditions L and N (z = 1.46) and L and S (z = -2.08). These results indicate that turn-length in the no delay condition is shorter than in the short delay condition, but that there is no difference between the other conditions.

### Gaze and sociality during delays

#### Q3: Is there a correlation between gaze behavior and perception of the robot’s social attributes (e.g. fluency, likability etc.)?

Spearman’s rank correlation analysis revealed a significant negative correlation between gaze to hand behavior frequency and NARS scores (Spearman’s correlation coefficient $\rho$ = −0.53, *p*<0.05, see Fig. 7c). Each data point in the correlation analysis represents a single participant’s average gaze to hand frequency and their corresponding average NARS rating, aggregated across all trials. The result indicates that participants with more negative attitudes towards robots tended to exhibit lower frequencies of gaze to hand behavior during the interaction. The negative $\rho$ value signifies a moderate inverse relationship, meaning as the NARS scores increase (indicating more negative attitudes), the frequency of gaze to hand behavior decreases. To examine whether this result was driven by a potential outlier, we reran the analysis with the extreme data point removed. The negative correlation remained significant ($\rho$ = –0.51, *p*<0.05). There was also a significant moderate negative correlation between face gaze duration and fluency ratings of participants ($\rho$ =−0.52, *p*<0.05, see Fig. 7d). This suggests that higher durations of face gaze are associated with lower ratings of fluency. To address concerns regarding a potential outlier in the correlation, we conducted a follow-up Spearman correlation analysis with the outlier removed. The effect remained and was slightly stronger ($\rho$ = –0.58, p<0.05), suggesting the association is robust, too.

There was a strong positive correlation between pre-interaction likability of the robot and duration of gaze to hand behavior ($\rho$ = 0.7, *p*<0.01, see Fig. 7e), indicating that the more likable the robot was rated initially, the more gaze to hand behavior was exhibited during the interaction.

Apart from that, there were no significant correlations between fluency, trust, post-interaction social attributes (as rated in the Godspeed Questionnaire) and any of the gaze behaviors.

## Discussion

This study aimed to investigate the relationship between timing and sociality in HRI by examining how delays affect participant gaze behavior and perceptions of Epi’s social features. The results show that participants adapt their gaze patterns during delays and that perceived sociality may be linked to gaze behavior.

Our assumption was that the impact of delays on human behavior should depend on their appropriateness, i.e. a short delay that could be interpreted as a thinking pause should lead to higher engagement and thus more gaze to hand, while a longer delay should lead to less participant gaze to hand behavior and higher face gaze since the participants might want to monitor ’what is going on’ with the robot.

Regarding overall gaze behavior differences across delay conditions (Q1a), we observed that the no-delay condition showed the highest gaze to hand behavior, followed by short and long delays. Post hoc tests confirmed significant differences between the no-delay and both delay conditions, but not between short and long delays. This suggests that the presence of any delay, regardless of length, reduces gaze to hand behavior, contradicting our hypothesis that short delays would foster more engagement. Face gaze duration did not differ significantly across conditions, though a marginal trend suggested more face gaze in the long delay and no-delay condition. In human interaction, people often look at others’ faces to find cues that help make sense of a situation and reduce uncertainty^26,47^. However, Epi’s face lacked informative cues in our study, which may explain the lower face gaze in the short delay condition. The fact that we found that participants were more likely to transition from gazing at the hand to face gaze than to return to the hand (and conversely, less likely to transition from face gaze to gaze to hand than to look at the face again) (Table 6), supports the idea that participants may have been searching for cues in the robot’s face, even though those cues were absent. For gaze away behavior, no significant condition differences were found in duration, but we observed a difference in frequency: participants looked away less often in the short delay condition than in the no-delay or long-delay conditions. This suggests that intermediate delays may reduce disengagement compared to both extremes. No significant changes in gaze behavior were observed across different match sequences.

Our hypothesis was confirmed regarding gaze patterns during delays (Q1b): gaze behavior changed when the robot hovered with its arm. This phenomenon however seems to be a local rather than global effect, considering that the Friedman test showed no significant results, while comparing the delay and non-delay periods for each match in the Wilcoxon signed-rank test showed significant differences. Bayesian analysis showed differences between delay and non-delay periods as well. Participants spent significantly more time looking at Epi’s face during delay periods compared to non-delay periods. This aligns with prior research suggesting that unexpected delays prompt participants to look at the robot’s face for explanatory cues^8,26^. However, this contrasts with our overall finding that face gaze was more frequent in the no delay condition, indicating that further investigation with a larger sample size may be needed. One explanation is that engagement temporarily increases during delays as a monitoring strategy, but then drops afterward, possibly due to fatigue. The duration and significance of face gaze remained consistent from the second to the eighth match, suggesting that participants did not stop looking at the robot’s face, despite it lacking informative cues. This may reflect an intuitive social impulse to seek meaning in the agent’s face, even when none is available. The lack of expressivity and communicative cues may have encouraged participants to focus more on Epi’s face, searching for any signal to interpret the situation. We also found significantly more gaze to hand behavior during delays compared to non-delay periods. While this contradicts the overall trend of higher gaze to hand in the no delay condition, it supports the idea that delays provoke compensatory monitoring behavior.

According to Rosen^22^, fluency and expectations are intertwined, where the latter determines the perception of the former; our results fit well into that explanation. Although we found no link between anthropomorphism and gaze behavior, increased face gaze during delays could indicate that the robot is being treated as a social agent. As artificial agents become more integrated into daily life, we are likely to develop new strategies for anticipating their actions^4^. As mentioned above, the intentional stance refers to our tendency to interpret others’ actions as driven by mental states. Depending on the stance taken, different expectations arise about how the agent should act^4^. In line with this, our findings for Q3 show that pre-interaction likability was correlated with gaze to hand behavior ($\rho$ = 0.699, p<0.01), suggesting that participants who perceived the robot as more likable were more engaged with its actions. Negative attitudes toward robots, conversely, correlated negatively with gaze to hand, indicating that skepticism leads to reduced engagement. These results imply that positive initial impressions can improve interaction quality, as reflected in gaze to hand behavior. We also found a moderate negative correlation between face gaze duration and fluency ratings ($\rho$ = -0.517, p<0.05), indicating that more face gaze was associated with lower perceived fluency. This may suggest compensatory behavior, where participants seek non-verbal cues in response to difficulties. No significant correlations were found between other social attributes, such as trust or post-interaction ratings, and any gaze behaviors.

Regarding our second research question, we cannot confirm or reject that participants adapted to the robot’s movements. While participants appeared to take longer turns during the short delay condition than in the no delay condition, there was no strong effect of match number, making sustained adaptation over time unlikely. One reason for not finding clear evidence of behavioral adaptation is that the interaction was highly structured. The Tic-Tac-Toe game is turn-based and constrained, limiting opportunities for dynamic timing adjustments. Moreover, Epi’s minimized social signals, to isolate the effect of movement delays, may have dampened participants’ social behaviors. Future studies could explore this in less structured, more naturalistic settings, as seen in Kose-Bagci et al.^25^. Another limitation concerns the relatively coarse delay intervals used in this study (4 and 10 seconds), which restrict our ability to generalize about the effects of delays falling between those values. It is possible that the perception of a delay as socially meaningful or intentional, rather than disruptive, occurs just above or below the 4-second mark, especially if the relationship between time/delay duration and human interpretation is non-linear, as indicated by various studies^48,49^. Future research should therefore investigate more fine-grained delay intervals to better pinpoint the threshold for behavioral adaptation.

We acknowledge that the small sample size (n = 17), due to the time-intensive video analysis and limited resources, affects the statistical power and limits the generalizability of our findings. These limitations may also be exacerbated by our between-subject design. The computed Cohen’s d figures (Table 4) indicate that gaze to hand (N vs. S) and gaze looking away (S vs. L) are of interest at $d \approx 1.30$ and $d \approx -0.85$, respectively. However, the confidence intervals (CIs) are larger than 1 in both cases, implying considerable uncertainty. As we observed interesting effects, it might be beneficial to replicate the experiment with a larger sample size.

As previously mentioned, Epi did not display gaze behavior or facial expressions in this study, as we aimed to minimize potential confounding variables. However, future studies could incorporate expressive cues like gaze to explore how nonverbal signals interact with delayed responses. For instance, prior research shows that participants interpret Epi’s emotional expressions through the color, direction, and light intensity of its eyes, offering a foundation for integrating gaze and delay behaviors^50^. Since gaze behavior may reflect cognitive processing demands^51^, it could be useful to include physiological measures of cognitive load, such as cardiovascular activity. Alternatively, game complexity and uncertainty could be monitored as indicators of cognitive demand. Another direction that could be explored in regards to our results is the ‘Out of the Loop (OOTL)’ phenomenon, which appears when humans are over-reliant on artificial or autonomous systems and thus fail to take control when errors happen. This phenomenon is one of the main risks when working in automatized environments, where humans fail to understand and predict the system’s next actions^4^. Given the observed increase in face gaze during delay conditions, correlated with lower fluency ratings, it would be interesting to explore whether this monitoring helps participants stay engaged and ready to respond to errors.

In this article we do not present any qualitative findings, however we observed differences in regards to how much participants seemed to be ‘in the loop’ and modified the robot’s actions when they deemed them faulty. Thus, future studies could benefit from incorporating post-interaction interviews to contextualize and enrich the quantitative findings from the questionnaires. It could also be beneficial to use an ethnographic approach, to investigate how each action sequence influences the next one, especially in regards to how humans make sense of the robot’s movements being delayed and how much control they allow themselves to take. This could be even further investigated in multi-party scenarios where several participants account for the robot’s actions. Pelikan & Hofstetter^9^ found that participants collectively manage delays by adjusting to the robot’s actions, legitimizing pauses, and supporting the robot’s performance through instructions and coordination.

## Conclusion

This study provides insights into how varying delay conditions affect human behavior during interactions with a humanoid robot. Contrary to our initial hypothesis, shorter delays did not enhance engagement through gaze to hand; instead, the absence of delay led to the most gaze to hand behavior. The finding that both short and long delay conditions equally diminished gaze to hand indicates that any delay, regardless of its length, disrupts engagement. Although face gaze duration did not vary significantly across conditions, increased face gaze during delays indicates participants monitored the robot during pauses. The correlation between pre-interaction likability and gaze to hand behavior, as well as the negative relationship between face gaze and perceived fluency, suggests that participants’ initial impressions and compensatory behaviors play a significant role in shaping the interaction.

These findings emphasize the importance of minimizing delays in human-robot interactions to foster engagement and natural behavior. Furthermore, the study highlights the potential for gaze behavior to serve as an indicator of participant attitudes and perceptions, providing opportunities for robots to adapt their responses in real-time.

## Supplementary Information

Supplementary Information.

## Data Availability

The datasets generated and/or analyzed during the current study are not publicly available due to the nature of the raw data making it impossible to ensure anonymity of the participants, and since Lund University imposes strict restrictions on data sharing.However, coded video files are available from the corresponding author upon reasonable request.

## References

[1] Breazeal, C. Toward sociable robots. *Robotics Auton. Syst.* **42**, 167–175 (2003).

[2] Fantasia, V. & Delafield-Butt, J. Time and sequence as key dimensions of joint action development. *Dev. Rev.* **69**, 101091 (2023).

[3] Rawls, A. W. & David, G. Accountably other: Trust, reciprocity and exclusion in a context of situated practice. *Hum. Stud.* **28**, 469–497 (2005).

[4] Ciardo, F., Tommaso, D. & Wykowska, A. Joint action with artificial agents: Human-likeness in behaviour and morphology affects sensorimotor signaling and social inclusion. *Comput. Human Behav.* **132**, 107237 (2022).

[5] Chao, C. & Thomaz, A. L. Timing in multimodal turn-taking interactions: Control and analysis using timed petri nets. *J. Human-Robot Interact.* **1**, 4–25 (2012).

[6] Rich, C., Ponsler, B., Holroyd, A. & Sidner, C. L. Recognizing engagement in human-robot interaction. In *2010 5th ACM/IEEE International Conference on Human-Robot Interaction (HRI)*, 375–382 (IEEE, 2010).

[7] Riek, L. D., Paul, P. C. & Robinson, P. When my robot smiles at me: Enabling human-robot rapport via real-time head gesture mimicry. *J. Multimodal User Interfaces* **3**, 99–108 (2010).

[8] Iqbal, T., Gonzales, M. J. & Riek, L. D. A model for time-synchronized sensing and motion to support human-robot fluency. In *ACM/IEEE International Conference on Human-Robot Interaction (HRI), Workshop on Timing in HRI*, 1–6 (2014).

[9] Pelikan, H. & Hofstetter, E. Managing delays in human-robot interaction. *ACM Trans. Comput. Human Interact.* **30**, 1–42 (2023).

[10] Tian, L. & Oviatt, S. A taxonomy of social errors in human-robot interaction. *ACM Trans. Human-Robot Interact. (THRI)* **10**, 1–32 (2021).

[11] Dragan, A. D., Lee, K. C. & Srinivasa, S. S. Legibility and predictability of robot motion. In *2013 8th ACM/IEEE International Conference on Human-Robot Interaction (HRI)*, 301–308 (IEEE, 2013).

[12] Hoffman, G. & Breazeal, C. Effects of anticipatory action on human-robot teamwork efficiency, fluency, and perception of team. In *Proceedings of the ACM/IEEE international conference on Human-robot interaction*, 1–8 (2007).

[13] Ragni, M., Rudenko, A., Kuhnert, B. & Arras, K. O. Errare humanum est: Erroneous robots in human-robot interaction. In *2016 25th IEEE International symposium on robot and human interactive communication (RO-MAN)*, 501–506 (IEEE, 2016).

[14] Salem, M., Eyssel, F., Rohlfing, K., Kopp, S. & Joublin, F. To err is human (-like): Effects of robot gesture on perceived anthropomorphism and likability. *Int. J. Soc. Robotics* **5**, 313–323 (2013).

[15] Chan, J. & Hwang, Y. Trust and forgiveness in service: effects of single and double deviations with human and robot staff. *Asia Pacific Journal of Tourism Research* (2025).

[16] Mirnig, N. et al. To err is robot: How humans assess and act toward an erroneous social robot. *Front. Robotics AI* **4**, 251625 (2017).

[17] Giuliani, M. et al. Systematic analysis of video data from different human-robot interaction studies: A categorization of social signals during error situations. *Front. Psychol.* **6**, 931 (2015). 26217266 10.3389/fpsyg.2015.00931 PMC4495306

[18] Zhou, A., Hadfield-Menell, D., Nagabandi, A. & Dragan, A. D. Expressive robot motion timing. In *Proceedings of the 2017 ACM/IEEE international conference on human-robot interaction*, 22–31 (2017).

[19] Okuno, Y., Kanda, T., Imai, M., Ishiguro, H. & Hagita, N. Providing route directions: design of robot’s utterance, gesture, and timing. In *Proceedings of the 4th ACM/IEEE international conference on Human robot interaction*, 53–60 (2009).

[20] Schoeller, F., Miller, M., Salomon, R. & Friston, K. J. Trust as extended control: Human-machine interactions as active inference. *Front. Syst. Neurosci.* **15**, 669810 (2021). 34720895 10.3389/fnsys.2021.669810 PMC8548360

[21] Desai, M., Kaniarasu, P., Medvedev, M. S., Steinfeld, A. & Yanco, H. A. Impact of robot failures and feedback on real-time trust. *2013 8th ACM/IEEE International Conference on Human-Robot Interaction (HRI)* 251–258 (2013).

[22] Rosén, J. Expectations in human-robot interaction. In *Advances in Neuroergonomics and Cognitive Engineering: Proceedings of the AHFE 2021 Virtual Conferences on Neuroergonomics and Cognitive Engineering, Industrial Cognitive Ergonomics and Engineering Psychology, and Cognitive Computing and Internet of Things, July 25-29, 2021, USA*, 98–105 (Springer, 2021).

[23] Baimel, A., Severson, R. L., Baron, A. S. & Birch, S. A. Enhancing, “theory of mind’’ through behavioral synchrony. *Front. Psychol.* **6**, 870 (2015). 26157415 10.3389/fpsyg.2015.00870 PMC4477228

[24] Robins, B., Dautenhahn, K., Te Boekhorst, R. & Nehaniv, C. L. Behaviour delay and robot expressiveness in child-robot interactions: a user study on interaction kinesics. In *Proceedings of the 3rd ACM/IEEE international conference on Human robot interaction*, 17–24 (2008).

[25] Kose-Bagci, H., Dautenhahn, K., Syrdal, D. S. & Nehaniv, C. L. Drum-mate: Interaction dynamics and gestures in human-humanoid drumming experiments. *Connect. Sci.* **22**, 103–134 (2010).

[26] Admoni, H. & Scassellati, B. Social eye gaze in human-robot interaction: A review. *J. Human-Robot Interact.* **6**, 25–63 (2017).

[27] Abele, A. Functions of gaze in social interaction: Communication and monitoring. *J. Nonverbal Behav.* **10**, 83–101 (1986).

[28] Ruhland, K. *et al.* A review of eye gaze in virtual agents, social robotics and hci: Behaviour generation, user interaction and perception. In *Computer graphics forum*, vol. 34, 299–326 (Wiley Online Library, 2015).

[29] Land, M. F. & Hayhoe, M. In what ways do eye movements contribute to everyday activities?. *Vis. Res.* **41**, 3559–3565 (2001). 11718795 10.1016/s0042-6989(01)00102-x

[30] Yu, C., Schermerhorn, P. & Scheutz, M. Adaptive eye gaze patterns in interactions with human and artificial agents. *ACM Trans. Interact. Intell. Syst. (TiiS)* **1**, 1–25 (2012).

[31] Schneider, B. & Pea, R. The effect of mutual gaze perception on students’ verbal coordination. In *Edm*, 138–144 (2014).

[32] Brinck, I. *et al.* Humans perform social movements in response to social robot movements: Motor intention in human-robot interaction. In *2020 Joint IEEE 10th International Conference on Development and Learning and Epigenetic Robotics (ICDL-EpiRob)*, 1–6 (IEEE, 2020).

[33] Ijuin, K., Jokinen, K. J., Kato, T. & Yamamoto, S. Eye-gaze in social robot interactions grounding of information and eye-gaze patterns. In *Proceedings of the Annual Conference of JSAI 33rd (2019)*, 3J3E402–3J3E402 (The Japanese Society for Artificial Intelligence, 2019).

[34] Stedtler, S. *et al.* Is there really an effect of time delays on perceived fluency and social attributes between humans and social robots? a pilot study. In *Companion of the 2024 ACM/IEEE International Conference on Human-Robot Interaction*, 1013–1017 (2024).

[35] Bakeman, R. & Quera, V. *Sequential analysis and observational methods for the behavioral sciences* (Cambridge University Press, 2011).

[36] Balkenius, C., Johansson, B. & Tjøstheim, T. A. Ikaros: A framework for controlling robots with system-level brain models. *Int. J. Adv. Robotic Syst.* **17**, 1729881420925002 (2020).

[37] Johansson, B., Tjøstheim, T. A. & Balkenius, C. Epi: An open humanoid platform for developmental robotics. *Int. J. Adv. Robotic Syst.* **17**, 1729881420911498 (2020).

[38] Syrdal, D. S., Dautenhahn, K., Koay, K. L. & Walters, M. L. The negative attitudes towards robots scale and reactions to robot behaviour in a live human-robot interaction study. *Adaptive and emergent behaviour and complex systems* (2009).

[39] Bartneck, C., Kulić, D., Croft, E. & Zoghbi, S. Measurement instruments for the anthropomorphism, animacy, likeability, perceived intelligence, and perceived safety of robots. *Int. J. Soc. Robotics* **1**, 71–81 (2009).

[40] Hoffman, G. Evaluating fluency in human-robot collaboration. *IEEE Trans. Human-Mach. Syst.* **49**, 209–218 (2019).

[41] Schaefer, K. E. Measuring trust in human robot interactions: Development of the “trust perception scale-hri”. In *Robust intelligence and trust in autonomous systems*, 191–218 (Springer, 2016).

[42] DeVries, R. & Fernie, D. Stages in children’s play of tic tac toe. *J. Res. Childhood Educ.* **4**, 98–111 (1990).

[43] Miller, R. B. Response time in man-computer conversational transactions. In *Proceedings of the December 9-11, 1968, fall joint computer conference, part I*, 267–277 (1968).

[44] Shiwa, T., Kanda, T., Imai, M., Ishiguro, H. & Hagita, N. How quickly should a communication robot respond? delaying strategies and habituation effects. *Int. J. Soc. Robotics* **1**, 141–155 (2009).

[45] Hoenig, J. M. & Heisey, D. M. The abuse of power: The pervasive fallacy of power calculations for data analysis. *Am. Stat.* **55**, 19–24 (2001).

[46] Abd, Al-Hameed Ali. & K,. Spearman’s correlation coefficient in statistical analysis. *Int. J. Nonlinear Anal. Appl.* **8**, 3249–3255 (2022).

[47] Hessels, R. S. How does gaze to faces support face-to-face interaction? a review and perspective. *Psychon. Bull Rev.* **27**, 856–881 (2020). 32367351 10.3758/s13423-020-01715-w PMC7547045

[48] Crystal, J. D. Nonlinear time perception. *Behav. Process.* **55**, 35–49 (2001). 10.1016/s0376-6357(01)00167-x 11390090

[49] Witowska, J., Zajenkowski, M. & Wittmann, M. Integration of balanced time perspective and time perception: The role of executive control and neuroticism. *Personal. Individ. Differ.* **163**, 110061 (2020).

[50] Tärning, B., Tjøstheim, T. A., Mirström, M. M. & Johansson, B. Expressing robot emotion using eye colors, pupil sizes, eye direction and head postures. *Int. J. Soc. Robotics* **9**, 1–13 (2025).

[51] Novick, D. G., Hansen, B. & Ward, K. Coordinating turn-taking with gaze. In *Proceeding of Fourth International Conference on Spoken Language Processing. ICSLP’96*, vol. 3, 1888–1891 (IEEE, 1996).

