# Supplementary material for: Gaze and movement adaptation in response to delayed robotic movement during turn-taking
Source: Sci Rep. 2025 Sep 30;15:34098. doi: 10.1038/s41598-025-17140-9 (PMC12485069; doi:10.1038/s41598-025-17140-9)
Supplement: Supplementary file 1 — Supplementary Information. [file 41598_2025_17140_MOESM1_ESM.pdf]

Appendix

Post-hoc results

Table 1. Gaze Duration Post-hoc results, reporting mean difference and significance by Condition. * p < 0.05, ** p < 0.01, *** p < 0.001.

Comparison	Gaze to hand behavior	Face Gaze	Gaze Away
N-S	-0.49***	-0.44	-0.66
N-L	0.31**	-0.003	-0.06
S-L	-0.17	-0.45	-0.72

Table 2. Gaze Relative Frequency Post-hoc results, reporting mean difference and significance by Condition. * p < 0.05, ** p < 0.01, *** p < 0.001.

Comparison	Gaze to hand behavior	Face Gaze	Gaze Away
N-S	0.40***	-0.35	-0.58*
N-L	0.21	0.11	0.08
S-L	0.19	-0.23	-0.50

Complementary Bayesian analysis

To supplement our primary analysis shown in the main text, we modeled the difference in time periods using Bayesian multilevel regression. The Bayesian analysis was done in brms v2.22.0 using the two following models:

Simple formula: $\text{Difference} \sim 0 + \text{Match}$

Complex formula: $\text{Difference} \sim 0 + \text{Match} + (1|\text{Participant})$

Initial analysis indicated the presence of outliers, particularly for "Gaze Away" behaviour. To avoid these outliers unduly biasing estimates, the models were fitted using a Student's-t distribution which down-weight outliers. For both models, the fitting process was run for 4000 steps. Model comparison using *loo_compare* shows that the simple model was the best fit for Gaze to Hand and Gaze Away behaviours, while the complex model was the best fit for Gaze to Face - implying that there was an effect of participants for the Gaze to Face condition (see table 3 for model comparison details and table 5 for model parameter estimates).

The difference is computed as $difference = out - in$; i.e. a negative value means time periods "inside delays" is larger than time periods "outside delays" as described in the main text. The analysis shows that for Gaze to Hand, the posterior distribution for the difference moves towards zero from match 2 to 5, but then becomes larger for match 7 and 8 (see figure 1, and table 4 for posterior summaries). For "Gaze Away" behaviour,

Table 3. Model comparison results from loo comparison.

Measure	Comparison	ELPD Difference	SE	Winning Model
Gaze to Hand	Simple vs. Complex (Participant RE)	-0.35	1.90	Simple
Gaze to Face	Simple vs. Complex (Participant RE)	-11.32	4.84	Complex
Gaze Away	Simple vs. Complex (Participant RE)	-1.29	1.35	Simple

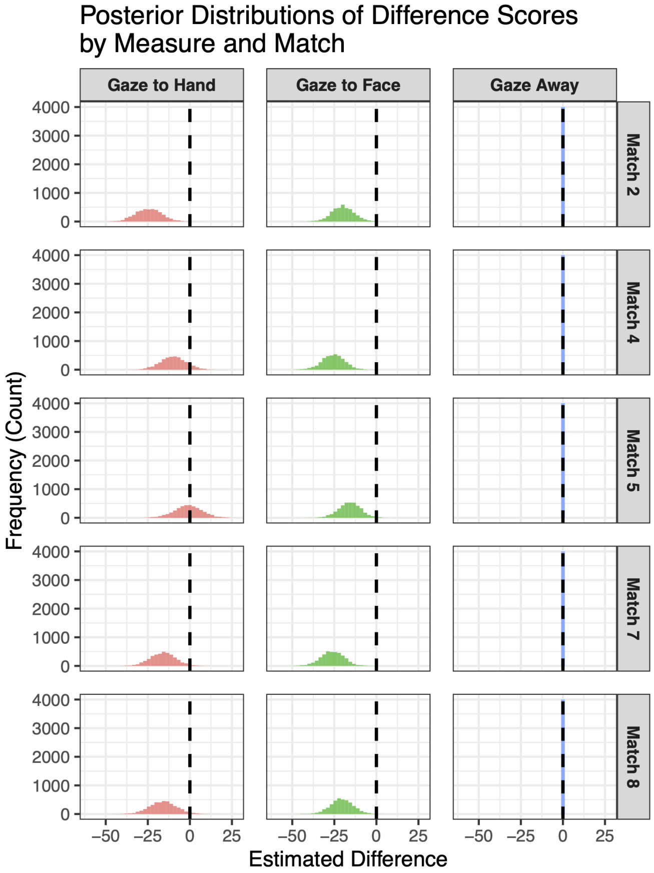

Figure 1. Posterior distribution of difference scores by measure and match. Difference initially reduces for Gaze to hand from match 2 to 7, but then increases for match 7 and 8. For Gaze to Face, difference stays negative - time inside delay is consistently greater than time outside dealys. For Gaze Away, there is no difference between inside and outside delay.

Table 4. Posterior summaries for the difference score, with lower and upper credible intervals (CrI).

Measure	Condition	Mean	Lower 95% CrI	Upper 95% CrI	P(Diff < 0)
**Gaze to Hand**					
	Match 2	-24.50	-40.21	-8.58	1.00
	Match 4	-9.79	-24.79	5.03	0.91
	Match 5	-1.08	-17.90	14.72	0.55
	Match 7	-15.75	-30.70	-0.75	0.98
	Match 8	-16.01	-31.89	0.16	0.97
**Gaze to Face**					
	Match 2	-19.93	-33.55	-6.06	1.00
	Match 4	-25.48	-39.12	-11.62	1.00
	Match 5	-15.82	-30.08	-1.44	0.99
	Match 7	-26.03	-40.44	-12.03	1.00
	Match 8	-20.69	-34.12	-5.79	1.00
**Gaze Away**					
	Match 2	0.04	-0.07	0.20	0.26
	Match 4	0.05	-0.11	0.27	0.28
	Match 5	0.03	-0.09	0.19	0.30
	Match 7	0.01	-0.25	0.26	0.43
	Match 8	0.06	-0.08	0.28	0.21

Table 5. Parameter estimates and 95% credible intervals (CrI) for the best-fitting model. For students-t distribution, *sigma* = estimated standard deviation, *nu* = degrees of freedom.

Parameter	Estimate	95% CrI
**Gaze to Hand: Fixed Effects**		
Match 2	-24.5	[-40.21, -8.58]
Match 4	-9.79	[-24.79, 5.03]
Match 5	-1.08	[-17.9, 14.72]
Match 7	-15.75	[-30.7, -0.75]
Match 8	-16.01	[-31.89, 0.16]
**Gaze to Hand: Distributional**		
sigma	24.24	[18.55, 30.54]
nu	19.38	[4.02, 53.68]
**Gaze to Face: Fixed Effects**		
Match 2	-19.93	[-33.55, -6.06]
Match 4	-25.48	[-39.12, -11.62]
Match 5	-15.82	[-30.08, -1.44]
Match 7	-26.03	[-40.44, -12.03]
Match 8	-20.69	[-34.12, -5.79]
**Gaze to Face: Random Effects**		
sd(Participant Intercept)	15.57	[8.2, 26.63]
**Gaze to Face: Distributional**		
sigma	14.72	[11.3, 18.91]
nu	23.19	[5.15, 57.88]
**Gaze Away: Fixed Effects**		
Match 2	0.04	[-0.07, 0.2]
Match 4	0.05	[-0.11, 0.27]
Match 5	0.03	[-0.09, 0.19]
Match 7	0.01	[-0.25, 0.26]
Match 8	0.06	[-0.08, 0.28]
**Gaze Away: Distributional**		
sigma	0.2	[0.09, 0.37]
nu	1.07	[1, 1.27]
